# How climate change skeptics (try to) spread their ideas: Using computational methods to assess the resonance among skeptics’ and legacy media

**DOI:** 10.1371/journal.pone.0240089

**Published:** 2020-10-05

**Authors:** Silke Adam, Ueli Reber, Thomas Häussler, Hannah Schmid-Petri

**Affiliations:** 1 Institute of Communication and Media Studies, University of Bern, Bern, Switzerland; 2 Centre for Media and Communication, University of Passau, Passau, Germany; Groupe ESC Dijon Bourgogne, FRANCE

## Abstract

We study the discursive resonance of online climate skepticism in traditional media in Germany, a country where climate skeptics lack public prestige and thus form a political counter-movement. We thereby differentiate two temporal dynamics: resonance can be continuous or selective, based on the exploitation of specific events. Beyond, we test whether such resonance is higher within the conservative media. We rely on news value theory to shed light on the mechanism facilitating or hindering such resonance and identify three indicators for resonance: frames, positions and actors. Using various computational methods as well as qualitative case studies, we examine the skeptical and traditional media discourses over a period of two years. Our analysis shows that there is no continuous resonance. However, our data reveal selective resonance: skeptics’ manage to exploit specific events pushing their frames and positions onto traditional media’s agenda. Thereby, conservative media did not give greater resonance to climate skeptical voices whereas they resort to downplaying the issue by allocating less space to it.

## 1. Introduction

Anthropogenic climate change is one of the most fundamental problems the world is facing, and it presents a threat to the global community [1]. Although there is scientific consensus that climate change is occurring, that the rise in temperature is predominantly due to human activity, that it has severe consequences for ecological systems, and that only a sharp reduction in CO2 emissions can limit climate change, political efforts have been lacking. One reason for the impasse is the counter-mobilization of climate skeptics [2], who have run campaigns that cast doubt on the scientific consensus and those responsible for it—the climate scientists. Climate skeptics and their spokespersons in the media and politics have been stunningly successful in the US, where not only conservative citizens forcefully reject climate politics [3] but also the Trump administration. By contrast, in most European countries those defending the climate consensus, the climate advocates, (still) dominate political institutions (e.g., [4]), traditional mass media (e.g., [5]), and public opinion (e.g., [6, 7]) with climate skeptics being in the role of counter-movements [8].

Such counter-movements, however, use another venue, the Internet, to push their ideas [9]. Here, climate skeptics are more active and visible compared to climate advocates in European countries, building connections with conservative media and transnational allies [10, 11], and even imposing their frames onto the agenda of climate advocates [12]. Yet, so far, research has hardly analyzed (for an exception, see [13]) whether there is any connection between climate skeptical online communication and the coverage in traditional media and politics (for this research deficit, see [11, 14]). The neglect of studying discursive resonance among venues is all the more surprising as research for the US convincingly shows that the discourse in the traditional channels influences public perceptions [3]: polarized media discourses lead to polarized public perceptions. Beyond, research so far has primarily focused on the US where climate skeptics are strong and prestigious. We, however, focus on climate skeptics as a political counter-movement and ask: How is the discursive resonance of online climate skepticism shaped in traditional media when climate skeptics lack public prestige?

This study seeks to make three contributions. First, our study connects venues that have mostly been examined separately, as research has focused either on climate skeptics’ online communication (e.g., [10, 11, 14]; for a summary, see [9]), or on their visibility in traditional media (e.g., [5, 15–17]), or on their strength in parliamentary settings (e.g., [4, 18]). By comparing the discourses across venues, we contribute to a better understanding of the conditions that lead to greater resonance of counter-movement ideas in mainstream discourses. Second, as climate skeptics are organized across national borders [2], we take into account the potential transnational flow of ideas, via the web, into national arenas. Finally, we apply computational approaches to the analysis of the core of political contests. They allow—in contrast to manual techniques—to study discursive resonance among different venues, outlets and over longer-time periods and thus grasp different temporal dynamics of resonance as well as different patterns of resonance among outlets.

To answer our research question, we first look at climate change skeptics as political counter-movement that lacks public prestige. We then turn to the theoretical mechanisms by which these political counter-movements may resonate with traditional media and then develop indicators to measure such resonance. Before turning to the results, we elaborate on the methods employed and elaborate why we focus on Germany. The paper concludes with discussing the implications of our study.

### 1.1 Climate skeptics as political counter-movement

Following Rahmstorf [19], climate skeptics either fully deny or cast doubt on the fact that global warming is taking place (trend skepticism), that humans are the main drivers of it (attribution skepticism), and/or that climate change is leading to severe consequences (impact skepticism). In addition, climate skeptics may also cast doubt on those producing scientific evidence (consensus skepticism, see e.g., [20]) or may question the relevance of binding policy regulations (policy skepticism, see e.g., [21, 22]). Previous research has shown that climate skepticism is often linked to certain worldviews and beliefs, such as conservatism and the support for free unregulated markets [23, 24]. Climate skepticism, however, is more than an individual attitude. Climate skeptics in the US have built what Dunlap and McRight [23] call a “climate change denial machine,” [23 p147] in which conservative politicians, media, and bloggers work hand in hand with think tanks and interest groups.

While climate skepticism has been characterized as a counter-movement [2, 25, 26]—or counter-coalition [11] based on its counter-status to scientific results [27]—the political status of climate skeptics varies greatly between countries. They are far from being a minority in the US, which has been cited as a country where climate change denial has progressed to a point of becoming the official presidential doctrine; but other countries such as Norway or Australia also show strong forms of climate skepticism (e.g., [6, 20]). However, in countries like Germany, climate change skeptics are more marginal. In such countries, they are *political* counter-movements in the sense of Fraser [8]: that are groups in society that put forward a minority position and, as a consequence, are excluded from the mainstream debate.

Unsurprisingly perhaps, most research on climate skepticism focus on the US case (for a critique, see [20])—the country in which climate skeptics are strongest and hardly excluded from mainstream debates. For the US, the climate change denial machine is well described [2, 13, 25, 28], with detailed findings on climate skeptics’ appearances in the media (e.g., [15, 29, 30]), in the (English-speaking) online world (e.g., [10, 14, 26]), and in politics (e.g., [18]). If we turn, however, to those countries in which climate skeptics are still political counter-movements, our knowledge is limited. While some research focused on appearances of skeptics in traditional media (often in comparison to the US; e.g., [5, 16, 31–33]), only few studies analyzed the presence of climate change skeptics in parliamentary arenas [4]. Other research has focused on skeptics’ online communication in these countries [9, 11, 12, 31]. Yet, to our knowledge, research has failed to study how separated the political counter-movement still is or whether we can observe discursive resonance between counter-movements debates (mostly conducted online) and mainstream discourses.

Research so far has remained primarily on a descriptive level, focusing on the degree of climate skepticism in different venues. Bringing these venues together seems all the more necessary as Fraser [8] has already pointed out that counter-movements follow two goals: Through their internal communication they develop a group identity, whereas their external communication is directed towards the mainstream public. Investigating the connection between climate skeptics’ online discourses and the wider public is all the more important as research has shown that climate skeptics fully exploit the affordance provided by the internet, regardless of their status in traditional arenas (e.g., [11, 12, 14, 26, 31]); they can bypass journalistic gatekeepers, and directly connect with like-minded others across national borders. However, whether their strength in online communication matters for other arenas is still to be shown. This requires the study of the intersection of discourses between different venues (for this desideratum, see also [16, 34]). Theoretically, thereby three scenarios are possible: if discourses remain separated, the public is fragmented into mainstream and counter-public. If discourses resonate which each other, it might well be that counter debates take up ideas of the mainstream. With the omnipresence of the mainstream debates, such resonance is quite likely. However, what is politically more relevant and thus of utter interest to us, is the more unlikely case that political counter-movements manage to resonate within the mainstream. Research clearly shows that such actors do not have routine access to established channels. Their success therefore depends largely on their resonance in the mainstream discourse [35–37].

### 1.2 Political counter-movements and mechanisms for media resonance

To better understand the mechanisms that lead to discursive resonance we employ a dual perspective that takes into account the activities of the climate skeptical counter-movement and the working routines of media coverage. Counter-movements such as climate skeptics seek public visibility to gain a voice in political debates and affect their outcome [8]. Even in hybrid communication environments [38], traditional media coverage still plays a crucial role in amplifying voices of actors, their positions and viewpoints. Being at a disadvantage *vis-à-vis* established actors, counter-movement actors rely on discursive strategies to pursue their goals, knowing that they fit all the better into the news cycle the more they adapt to the narrative needs and working routines of the media [39].

According to news value theory, journalists evaluate events based on specific professional selection criteria—the news factors—with regard to their worthiness of publication and prominence in the coverage (e.g., [40–43]). In the case of climate change, one of the strategies pursued by climate skeptics is to voice doubt about fundamental aspects such as climate science, as this ties readily to the news factor “conflict”, increases the newsworthiness of the coverage and thus their chance of being included in news reports [15]. It has already been argued that the news factor “conflict” eases the resonance of skeptic ideas within mainstream journalistic debate [15]. Beyond this, we may argue that the news factor “surprise” pushes skeptics’ ideas onto the agendas. The downside of this strategy is that it risks losing much of its newsworthiness after a while, unless climate skeptics succeed in introducing new, unexpected perspectives to the debate that emphasize the news factor “surprise”. Finally, climate skeptics can attempt to rely on the prestige of some of the members of the counter-movement and thus emphasize the news factor “status/elite”.

In countries where climate skeptics are still a minority movement and do not command the necessary status, getting media visibility is less likely for them. This prestige factor is all the more important as research strongly shows that legacy media have a bias towards the elites [35, 36]. With regard to climate change skeptics, we can thus ask:

(RQ1): How is the discursive resonance of online climate skepticism shaped in traditional media coverage when climate change skeptics lack public prestige?

As we have seen above, news value theory suggests that the more news factors are attached to an event or issue and the stronger they are, the higher the chance that it is taken up by the media. Thereby, media coverage is driven by two different temporal dynamics. First, some news factors grant continuous media resonance. This is most likely the case for actors commanding a high level of prestige as the news factor “status / elite” describes a continuous (social and) discursive quality. Furthermore, prestigious actors on the climate skeptical side would allow the counter-movement to promote the position and their perspectives. Second and in contrast to this, counter-movements might rely on the creation of specific events—or their exploitation, to generate selective media resonance. The news factor “surprise” is clearly related to this discursive strategy. We are thus interested which types of resonance we observe in countries where climate skeptics lack prestige and thus ask:

(RQ2): Is there rather a continuous or a selective congruence between the online communication of climate skeptics and traditional media coverage?

Finally, researchers have started to question whether news factors are perceived similarly among different news outlets (e.g., [43]). This so-called “two-component” theory assumes that news factors as characteristics of events are perceived differently by journalists depending on the political orientation of the outlets. In the US for instance conservative media ascribe greater news value to the ideas of climate skeptics [23, 26, 32, 44]. This conservative alliance structure has also been shown for countries in which counter-movements still have a minority status. Here, specific conservative newspapers publish skeptics’ claims [5], and skeptics closely connect via hyperlinks to these right-wing media outlets [11]. Consequently, our study examines the following question:

(RQ3): Is there a special connection with regard to the discursive resonance between climate skeptics and conservative media?

### 1.3 Political counter-movements and indicators for media resonance

To determine the degree of congruence between the online communication of the climate skeptical counter-movement and traditional media coverage, and how it develops over time, we distinguish three different discursive dimensions [45]: (1) issues and frames, (2) the positions articulated, and (3) the visibility of actors. The more we see frames, positions and actors converge between the two venues, the more we can speak of a discursive resonance. If we see that skeptic frames, positions and actors resonate with the mainstream, the plausibility is high that the counter-movement has succeeded in spreading their ideas; whereas if frames, positions and actors from the mainstream turn more prominent in skeptics’ discourse, the opposite is the case. However, the methods employed in this paper, do not allow us to draw any direct conclusion as to causal direction.

#### Skeptical issues and frames

Following Fraser [8], counter-movements are excluded from mainstream debate. Consequently, one of their central goals is to make their issues and frames visible to a wider public. From a classical agenda-building perspective [46] this means that counter-movements seek to raise awareness of those issues that are important for them and try to frame debates from their viewpoint. In well-established issues, where the agenda is largely set by traditional media and political institutions, counter-movements primarily seek to re-frame the debate by promoting alternative views on an issue such as climate change. They attempt to shift “central organizing idea[s] or story line[s] that provide […] meaning to an unfolding strip of events […] The frame suggests what the controversy is about, the essence of the issue” [47 p143].

On the thematic level, climate skeptics do this by sowing doubt where there is (scientific) consensus, with the aim to draw climate advocates into a debate where contrarian positions might gain the upper hand [48]. Studies for the US [13] have shown that a positive semantic relation between climate skeptic’s online communication and traditional media coverage may occur, documenting the responsiveness of the media to contrarian ideas and thus the success of this strategy. In this perspective, an increase of skeptics’ frames on the media agenda might be taken as an indicator for discursive resonance independent of the fact, whether media counter-argue these frames or not. Pure visibility of frames matters [49].

#### Skeptical positions

Frames are only part of the discursive structure of a debate; equally important is the question whose position is being covered by the media. After all, climate skeptical frames can just as well be reported from a critical viewpoint that effectively undermines their credibility. When for instance climate skeptics succeed in provoking a debate about the uncertainty of scientific results (which is a classic skeptics’ frame, see [12]), mass media may decide to give voice to mainstream scientists who contest this frame. Similarly, journalists might not follow climate skeptical framing, but include their counter-position.

#### Climate skeptical participants in debates

In addition to frames and positions, counter-movements attempt to promote specific representatives of their cause, in an attempt to expand the range of legitimate participants in the debate. These actors gain visibility in two ways, either as speakers or addressees. In the first case, they may be spokespersons who relate the view of a skeptical think tank. This form of visibility is closely associated with the positional dimension introduced above. In such a setting, journalists use the counter-movement’s representatives as “opportune witnesses” [50] to make climate change skepticism more prominent. Second, climate skeptics may gain visibility as objects of reporting. In this role, they are ratified by other actors positively or negatively. Yet, in both instances, the counter-movement succeeds in gaining visibility in mainstream debates, which helps turn it into a legitimate actor.

Table 1 shows the different dimensions of climate skeptical discourse. To simplify the analytical framework, we distinguish four core types according to the thematic and positional dimensions. We use the actor dimension to further specify the typology.

**Table 1:** Types of discursive resonance.

		Frame resonance	
		Yes	No
Positional resonance	Yes	**Full resonance:** Counter-movement frames and positions become more central in the mainstream coverage	P**ositional resonance**: counter-movement positions become more central in the mainstream coverage
	No	Frame **resonance**: Counter-movement frames become more central in the mainstream coverage	**No resonance**: Counter-movement discourse remains marginal in the mainstream coverage

If frames and positions of counter-movements are mirrored in the coverage of mainstream media, we speak of full resonance. Pure positional resonance occurs if only the positions of counter-movements are reported, yet within the mainstream discourse. For climate skeptics, this would, for example, mean that their skeptical positions receive attention within the larger mainstream debate about the role of renewable energy in the transition from fossil fuels to a greener society. This indicator looks for the pure attention of skeptical positions without taking into account whether journalists counter-argue. However, research points out that visibility matters independent of the evaluations surrounding it [49]. By contrast, we speak of pure thematic congruence if the frames promoted by counter-movements become more prominently part of the relevance structure of the mainstream debate around climate change without, however, finding a parallel increase in their positions. Such a setting occurs if climate skeptical frames such as the credibility of scientific studies, receive attention in mainstream debates, though the skeptical position associated with it is dismissed. The media would cover the perspective promoted by climate skeptics but explicitly reject it, arguing for instance that climate science is credible, and scientific results confirm the trends established by previous research. Finally, if neither skeptical counter-movement frames nor positions resonate in the media, the counter-movement discourse remains segregated from the mainstream [8].

Full, positional and thematic resonance can be further classified according to whether skeptical actors gain visibility in the coverage and thus are ratified as legitimate participants in the debate. The more climate skeptical actors become visible together with their position and/or their frames, the more they succeed in becoming part of the debate on their own terms. Conversely, the less climate skeptical actors are mentioned in the coverage together with frames and/or positions, the more the debate is shaped by journalist and other actors such as mainstream politicians.

## 2. Case selection, data, and methods

### 2.1 Case selection

To study the discursive resonance between the (potentially transnational) online communication of climate skeptics and traditional media, we focus on Germany. Germany represents an ideal case as climate skepticism was a small but significant phenomenon in traditional media [51], parliamentary arenas [4, 52], and public opinion [6, 20] during the period of analysis (June 2012 –June 2014). However, this wide-spread acceptance of man-made climate change does not mean that there was an equally high level of agreement on political measures. Anyhow, our design allows to search for initial resonance increase among venues. Thereby, research for Germany also shows that climate skeptics successfully exploit the online world; although fewer in number, climate skeptics are more visible and more active online and more strongly connect to transnational allies compared to climate advocates [11].

In such a setting, it is possible to observe whether and how ideas from the political counter-movement may flow into mainstream debates, whereas such flows of ideas are hard to detect in those countries where climate skeptics are not a minority movement anymore as their ideas are already visible in all channels. The German setting, thus, may allow us to understand the spread of ideas, respectively, the conditions under which policy monopolies are destructed [53] by redefining the issue, by changing the positions formulated, or by changing the participants of the debate. This is even more interesting as some researchers have claimed that climate skeptics seem to be gaining attention and terrain in Germany: Brunnengräber [54] posited that climate skepticism is increasingly gaining societal acceptance, and Schmid-Petri and Arlt [55] show that climate skeptical arguments have slightly increased in mainstream German media over recent years.

### 2.2 Collecting data on the counter-movements’ communication

To study skeptics’ communication, we studied their online communication—the field in which they are most active. Hereby, we relied on hyperlink issue networks that originate from prominent counter-movement actors. We thereby followed the logic of snowball sampling—a method employed if researchers have limited knowledge about the overall population. Such method allows to detect also those skeptics that are less-known.

**Fig 1:**
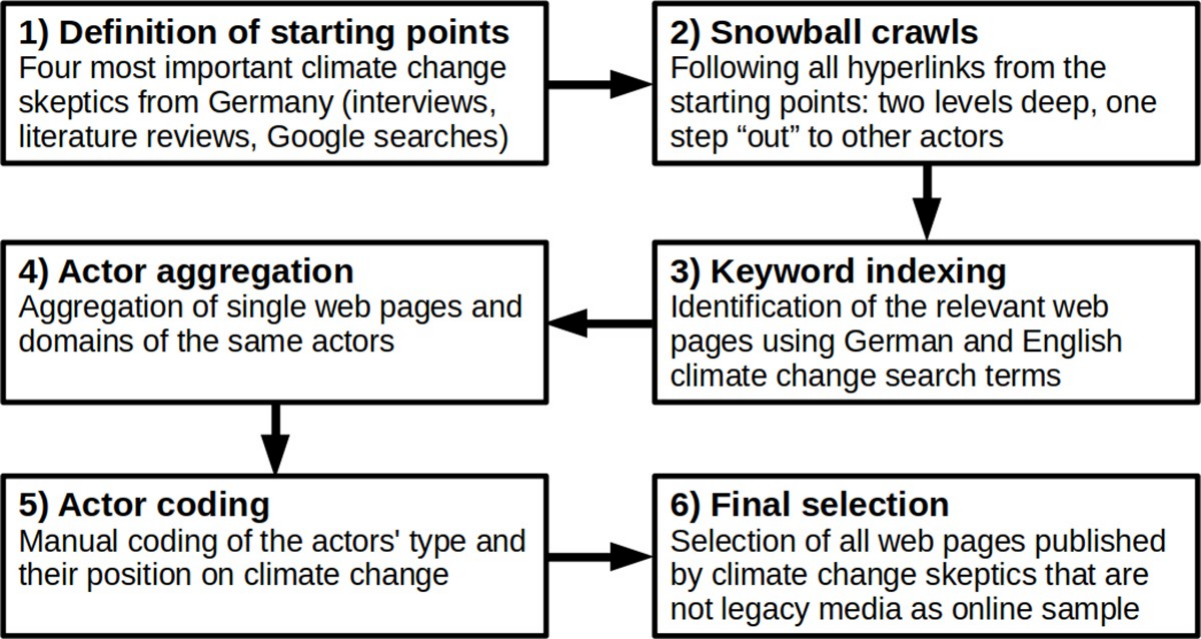
Online sampling procedure.

To identify relevant skeptics, we relied on a six-step procedure shown in Fig 1. First, we selected the four most important civil society actors of the climate skeptical counter-movement in Germany as starting points based on expert interviews, literature reviews, and country-specific Google searches (with deleted search histories). These are Analyse+Aktion, EIKE—Europäisches Institut für Klima und Energie, Klimaskeptiker, and Klimaüberraschung. We chose civil society actors as they show the broadest linking behavior [56] and because they are the “champions of online communication” [9 p530]. Second, starting from the actors’ main climate pages (list of URLs in S1 Appendix), crawling software (called Issuecrawler [57]) collected all hyperlinks two levels deep within the websites and all of those that pointed to other websites. We limited our snowball crawling to go only one step “out” as pre-studies have shown that further crawling substantially increases the number of pages that do not deal with climate change. Third, to make sure that only pages that were relevant to the climate debate remained in our network, we indexed all pages according to our keywords (i.e., “Klimawandel,” “globale Erwärmung,” “globaler Erwärmung,” “globalen Erwärmung”). We only indexed content that was publicly available, i.e. not password-protected. Also, we respected the robots exclusion standard (robots.txt). This standard allows website owners to define areas of their website that should not be scanned or indexed by robots (e.g. search engines, web crawlers). In this way, we made sure that the content was permitted for download by the website owners.

Fourth, some data preparation was necessary. We aggregated single web pages and domains that belonged to the same actors. This allowed us, in a fifth step, to attribute a position on climate change and a type of actor to each of the identified actors. To do so, we applied a manual content analysis, based on information found on the “About us” pages or similar sections of the website and conducted by two trained coders. They distinguished, position-wise, between climate skeptics, climate advocates, and climate neutrals (with no clear-cut position); separated legacy media from other actors; and coded their scope/country of activity (distinguishing a total of 199 geographical areas). Note that according to our definition, a climate skeptic is someone who explicitly questions at least one of the following: the existence of climate change, the human contribution to it, the science of climate change and/or its findings, projected trends/consequences of climate change, and/or the adaptation to it. By this definition, a climate skeptic is also someone who endorses the science but negates, for instance, its political and economic consequences. In turn, a climate advocate explicitly supports at least one of these points without doubting any of the others. The actors’ attributes were classified by two trained coders. The reliability of their classification was assessed by comparing them with a master coding and was calculated using Krippendorff’s alpha, which is a common statistical measure of the agreement between different coders when coding the same texts. The agreement is usually measured on the level of individual variables. The test revealed satisfactory results with Krippendorff’s Alpha of .90 for the position variable, .90 for the actor type variable, and .93 for the scope variable. In the final step, only those actors and their communication that put forward climate skeptical positions and were not rated as legacy media remained in our sample, which serves as a proxy for climate skeptics’ communication (154 different actors with a total of 13,009 unique web pages). Climate skeptics’ online communication here originates from prominent German climate skeptics from civil society, but then it is extended by snowball sampling to include text material from all types of skeptical non-legacy-media actors. As shown in Table 2, however, non-profit civil society actors and individual private persons account for the vast majority of the web pages in the final sample. Out of the 154 different actors, 33 are domestic actors from Germany with a primarily national scope, and 121 are foreign actors (either with a foreign national or transnational scope).

**Table 2:** Key figures of the skeptics’ online sample.

		Actors	Webpages
Domicile	German actors	33	3’549
	Foreign actors	121	9’490
Type	Politicians, political actors	3	3
	Socioeconomic pressure groups, companies	4	22
	Non-profit civil society actors	45	3’779
	Genuine online media	26	216
	Citizens / private persons	74	8’977
	Other actors	2	12
Total		154	13’009

We collected such online communication data of climate skeptics once a month over a period of two years (2012–14). We are aware that this proxy captures only part of climate skeptics’ communication, omitting, for example, online communication via social media (e.g., [58]). However, as actors are active on all venues and as research has shown that hyperlinking, i.e. referring to another actor by linking to its web presence, is closely related to social media and the interactions there [59], we are confident that such a partial approach might capture the relevant content of skeptics’ online communication (see for a similar argument, [60]).

### 2.3 Collecting data on mainstream debates in traditional media

We regard the national print media landscape (whether online or offline) as a good proxy for the mainstream debate. Research has shown that mass media have an elitist focus [35, 36] and, as such, are likely to reflect the mainstream debate. For Germany, we have selected the 15 most important daily and weekly newspapers as well as magazines with a national audience reach (see S2 Appendix). All of these outlets are regarded as national opinion-leaders. Within these outlets, we identified all relevant articles on climate change by searching for the above-mentioned keywords in the databases of Factiva and LexisNexis. This resulted in 4,111 articles about climate change in the observed period in German legacy media.

To find out whether discourse resonance is especially strong as regards the conservative media landscape, we finally classified the newspapers and magazines in our sample according to their ideological position. Following the work of Beck [61 pp153-155], Begenat [62 pp98-99], Lüter [63], Maurer and Reinemann [64 pp129-130], Pew Research Center [65], Schwarz-Friesel [66 p52], and Wessler and Rinke [67 p640], we identified the following nine legacy media as right of the center: *Bild*, *Bild am Sonntag*, *Die Welt*, *Frankfurter Allgemeine Zeitung*, *FAZ am Sonntag*, *Financial Times Deutschland*, *Focus*, *Handelsblatt*, and *Welt am Sonntag*. As shown in Table 3, they account for 1,495 articles in our sample (see S2 Appendix for exact number for each outlet). However, it is important to note that unlike in other countries (e.g., Great Britain), there are no hard right-wing newspapers in Germany, as even the most pronounced right-leaning paper (i.e., *Die Welt*) mostly adheres to liberal-pluralist principles [61].

**Table 3:** Key figures of the offline sample.

		Outlets	Articles
Type	Conservative media	9	1’495
	Other media	6	2’616
Total		15	4’111

The raw data collected from legacy media as well as from the counter-movements’ online communication is available to all interested researchers upon request via the open repository of GESIS (https://dx.doi.org/10.4232/1.5183). The R code produced for the data collection as well as for the analyses is publicly available on GitHub (https://github.com/ikmb-unibe/coab_so2).

### 2.4 Measuring discursive resonance

Our analysis is based on digital text on climate change of the skeptical counter-movement (online) and of legacy media (offline) over the course of two years. In a first step, we used a quantitative approach to check whether the resonance of the skeptics in the media has grown continuously. To do so, we aggregated the data on the level of single months and examined the discursive resonance using different computational methods. We acknowledge that this approach only captures the monthly correlation of the agendas and we therefore make no claims as to any strict causal relationships between counter- and mainstream-arenas. In a second step, however, we used a qualitative approach to reveal whether discursive resonance pushes skeptics’ frames, positions and actors occasionally. Of course, such patterns of increased discursive resonance may well be caused by third, unobserved factors, which play an important role in creating opportunities of discursive resonance. Such factors are hard to find in the continuous analysis whereas it is one strength of our qualitative analysis of the documents that such factors are detected. Table 4 provides an overview of the analytical techniques applied.

**Table 4:** Methods and techniques used to measure discursive resonance.

Resonance form	Approach	Discursive indicator	Method	Measure
Continuous	Quantitative	Frames	Topic model (STM, unsupervised): identification of frames used in documents	Jensen-Shannon divergence (ordinary least squares regression)
		Positions	Classifier (SVM, supervised): categorization of sentence as advocate, skeptical, or irrelevant	Difference of skeptical sentences rates on-/offline (ordinary least squares regression)
		Participants	Named-entity recognition (supervised/manual content analysis): identification of (non-)skeptical actors	Difference of skeptical actors rates on-/offline (ordinary least squares regression)
Selective	Qualitative	Frames, Positions, Participants	Identification of critical moments based on quantitative indicators, followed by an in-depth study of the factors triggering such discursive resonance.	

In order to detect continuous resonance, we employed a variety of computational methods and quantitative procedures. We used a bag-of-words *topic model* to detect shifts in the framing competition, employed a purpose-trained *classifier algorithm* to distinguish positions on climate change, and relied on a semi-automated *named entity recognition* procedure to show which actors participated in the debate. In the following paragraphs, we will shortly introduce each of these methods.

To identify *frames*, we relied on *probabilistic topic modeling* [68], an unsupervised method of automated content analysis [69], that helps unravel latent or hidden thematic structures of text material using Bayesian statistics. Topic models are mixed-membership models, meaning that every document consists of a mixture of different topics. Each document can therefore be understood as probability distribution over a set of topics and is best described by those topics with the highest probabilities. A topic, on the other hand, is defined by a “probability distribution over the entire corpus’ vocabulary” [70 p97] and represents a latent pattern of word (co-)occurrences. Those words that have a high probability within a topic are the ones that define it thematically.

In order to become meaningful, topics must be interpreted in a qualitative process and against the backdrop of a theory. Depending on both the corpus and the theory, the resulting thematic structures (i.e., probability distribution over words) may either be interpreted as actual topics (e.g., environment versus economy), as issues (e.g., climate change), or as frames (e.g., the emphasis on the scientific consensus), depending on the underlying type of text corpus [71]. As explained before, we understand frames as “central organizing idea[s] or story line[s]” [47 p143] that represent the “particular ways [in which] issues are presented” [72 p184]. In combination with the fact that we have keyword-cleaned texts, all dealing with climate change, and thus a relatively coherent corpus, we consider it valid to interpret the resulting latent patterns of word (co-)occurrences as frames or interpretative packages (see also [73]).

For our analysis, we rely on the commonly used and well documented structural topic model framework (STM; [74, 75]). As we removed duplicate web pages to compute our STM, it is based on a total of 17,120 text documents. This was done to avoid a bias in favor of the duplicated documents. As topic models only work with monolingual text material, we translated all the vocabulary of the English web pages into German before calculating the model [76]. We applied several common preprocessing steps in order to extract as much information from the corpus as possible. This included the removal of punctuation, conversion to all lowercase, removal of words with less than three characters, removal of stop words (e.g., “und”, “oder”, “auf”, “der”), stemming, and the removal of words that appear in less than 0.5% and in more than 99% of all documents (relative pruning). To decide on the number of topics (K), we combined data-driven indicators with a qualitative assessment of the interpretability of different solutions [70]. To do so, we first calculated 8 models with 5 to 40 topics. We then compared these models based on standard measures (i.e., held-out likelihood, semantic coherence, residuals). The models with 20 and 30 topics were selected for the final interpretation step. Based on both the topic top words and particularly relevant documents, the topics were interpreted by two people. The guiding question was whether top words and documents represent an interpretable and coherent frame. If both persons came to the same conclusion, the topic was labelled, otherwise it was excluded from the analysis. In the end, we chose the model with 20 topics, whereby five uninterpretable topics were excluded and two similar topics were merged (all labels and top words are shown in S3 Appendix). The calculation was done in R using the *stm* package [77].

To compare the similarity of frames used by climate skeptics in their online communication and in mass media reporting, we relied on the Jensen-Shannon divergence (JSD). This is a smoothed and symmetric derivative of the Kullback-Leibler (KL) divergence, which is a common measure when comparing distributions [78]. The normalized outcomes of the JSD can be used as measure of similarity between two probability distributions and is therefore well suited for the comparison of the topic distributions of our online and offline samples. A JSD of 0 would indicate complete congruence of the two distributions (i.e., the same frames used by the climate change skeptics and the legacy media). A JSD of 1 would mean completely different distributions and thus completely different frames. If climate skeptics succeed in influencing the thematic relevance structure of legacy media in a continuous way, we would therefore expect a declining JSD over the course of our two-year period of analysis. To detect whether there is a significant trend, we used ordinary least square regression following the approach taken by Farrell in his study on the influence of the climate change counter-movement [13]. Despite its relative simplicity, we find a linear model well suited to detect continuous resonance as the underlying theoretical idea is that of long-term discourse convergence.

To identify *positions*, we relied on a trained *classifier algorithm*, or, more precisely, on a linear support vector machine [79]. This classifier algorithm was used to categorize each single sentence in our documents as either advocate-leaning, skeptical-leaning, or irrelevant. In contrast to topic modeling, a classifier follows the logic of automated supervised content analyses [69]. This means that it follows the logic of a pre-defined coding scheme. In a first step, this coding scheme guides a manual content analysis of a text sample, distinguishing skeptical from advocative sentences. In a second step, the manually coded material serves as learning material for the computer algorithm [80]. To account for the two languages in our data set we trained two models, one for English documents and one for German documents.

To train the models, we used an active learning scenario. This means that we trained and checked the two models in several iterations, using manually coded sentences as training material and benchmark. The initial training set consisted of sentences from advocates and skeptics as well as of sentences that have nothing to do with climate change. The inclusion of such random sentences is crucial in order to be able to detect also irrelevant sentences in the data set. This initial training sets were used to train a first model for each language that was then applied to 10’000 random uncategorized sentences of our corpus in the respective language. After this first classification, the result was evaluated by a team of three human coders. The evaluated sentences were then added to the initial training data and the models were trained once again (hyperparameter C optimized by cross validation to avoid overfitting). We repeated this process three times, until we could not improve the classifiers’ performance anymore (measured by k-fold cross validation). To measure the accuracy of the classifiers, we treated the manually coded sentences as gold standard and compared them with the machine coded sentences. After the third iteration, the overall accuracy measured by the harmonic mean of precision and recall (micro-average) was F1 = 0.83 for the English model and F1 = 0.85 for the German model (see S4 Appendix for macro-average F1 of different categories). These are satisfying accuracy values. Classification was done in R using the LibLineaR package [81].

To assess the discursive resonance as regards positions, we analyzed whether the increase in skeptical sentences online is correlated with the share of skeptical sentences in the media. To do so, we first compare the share of skeptical sentences online and offline and study whether the differences of these shares increases or decreases. To detect potential trends, we again use linear regression models.

Finally, to identify *skeptical actors* within the debate, we relied on *named-entity recognition* (NER). It is a set of procedures to extract categories, like people’s names, organizations, and locations, from unstructured texts (for a short description, see [82]). To identify all actors in our sample of climate change web pages and media articles, we first used a list of around 1.3 million known named-entities as a look-up list (a lexical approach). On the one hand, the list consisted of names of prominent individuals (e.g., “Angela Merkel”) and multi-word units (e.g., “market economy”) that were not necessarily related to the climate change issue. On the other hand, it contained 10,095 names of actors that we had identified as important for the public discourse on climate change using manual content analysis [83]. In order to identify actors who were not on the list, we used two probabilistic sequence classifiers (English and German), which we trained specifically for this purpose using the list. We used conditional random field models from the Stanford CoreNLP package [84] as classifier algorithms.

To show discursive resonance as regards debates’ participants, we needed to add information to the named entities extracted. Thus, we relied on the list of 10,095 manually coded actors. For each actor, we coded the position on climate change*—*more precisely, whether the actor thinks that climate change is occurring and whether he/she sees it as a problem. This allowed us to identify the most important climate skeptics in our corpus. Using Kripendorff’s alpha again, the reliability scores were 0.69 for the first variable (occurrence of climate change) and 0.75 for the second (climate change seen as problem). This was measured as master-coder reliability based on a random sample of 30 actors. Both manually coded variables achieved satisfactory reliability scores.

Using these procedures, we identified a total of 46,901 skeptical actors, 443,452 advocative actors, and 411,955 actors without a clear position on climate change in our corpus. As before, we analyzed whether an increase in mentions of skeptical actors online was associated with an increase in mentions in the media. Again, we first compare the shares of skeptical actors used by both the skeptics and the legacy media. We then use the difference of the shares as divergence measure. Simple linear regression models are used to check for a significant convergence or divergence respectively.

To detect selective resonance, we used a qualitative approach. In a first step, we identified critical moments in the time series obtained by the computational procedures described above. Critical moments are points in time when the frames found online and offline were more similar than usual, when there were an increase of skeptical sentences in the media coverage, or when an unusual number of skeptical actors were mentioned in offline reporting. Once we identified such a critical moment, we examined the exact frame agenda in the offline corpus in this month and then determined the frames responsible for the increased similarity of the agendas. This means that we searched for frames that received an unusually high amount of attention in the legacy media’s reporting during this month. In a second step, we used this information to search for documents in our offline corpus in which the particular frames have a high proportion (>0.6). At the same time, the documents had to show a high number of skeptical actors or skeptical sentences. In this way, we identified documents with a high chance of selective resonance. In a third step, we looked at each of these documents as a separate case and tried to identify and describe both moments of selective resonance and factors causing it through in-depth reading. In the final step, we synthesized the knowledge gained from the case studies and put them into the wider context of legacy media coverage on climate change.

## 3. Results

**Fig 2:**
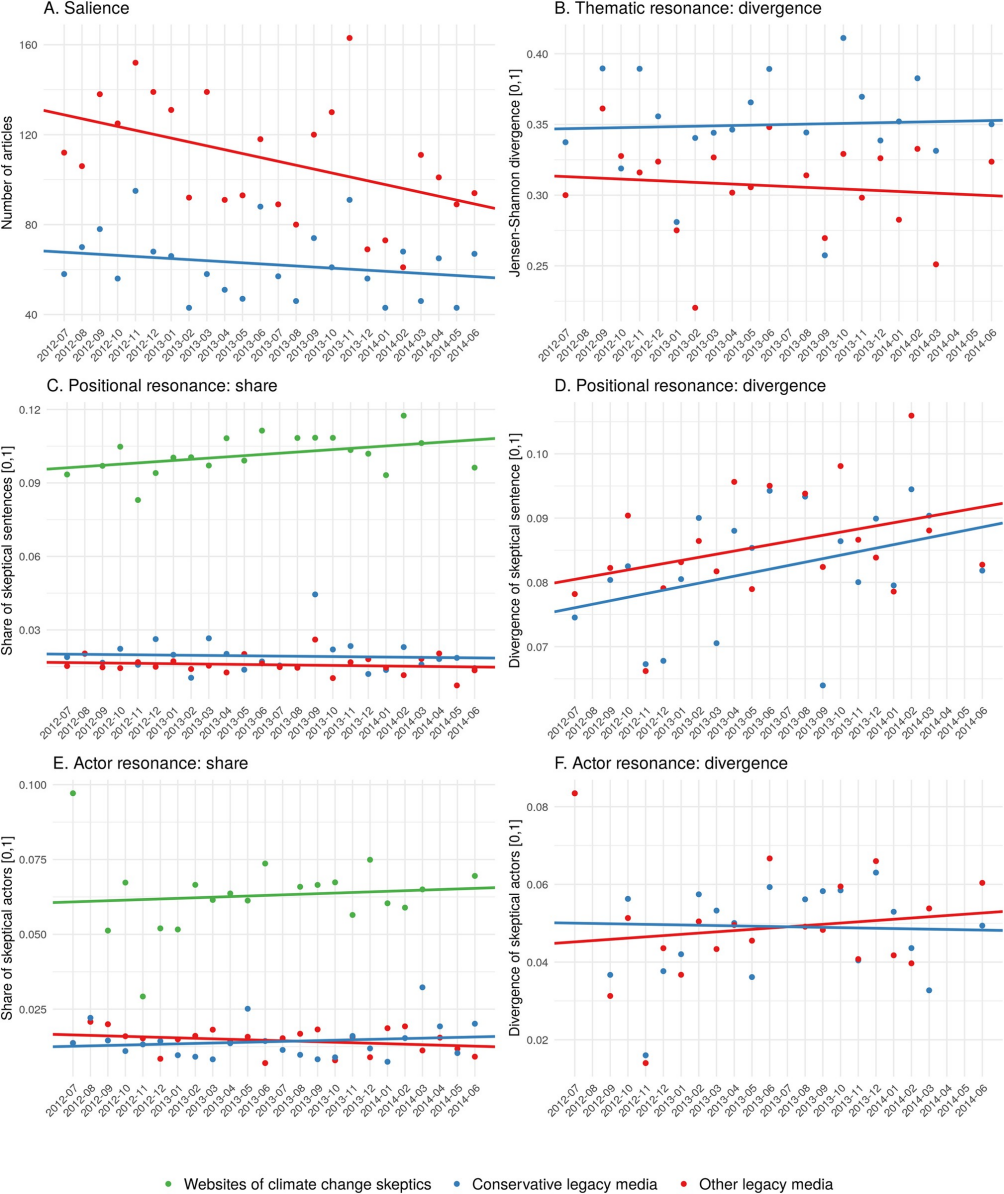
General salience of climate change and resonance of skeptical discourse for the period from July 2012 to June 2014. Plots (A-F): Salience of climate change in legacy media (A), divergence measures (B, D, F), share measures (C, E). Shown are the separate measurements for the websites of climate change skeptics, conservative legacy media, and other media (points) as well as the trend line for each of the three categories (lines). A trend line with positive incline indicates an growing number of articles on climate change (A), growing share of skeptical actors (C), growing share of skeptical actors (E), growing thematic divergence (B), growing positional divergence (D), growing divergence regarding the visibility of skeptical actors (F).

As a basis for our further analysis of the three indicators for discursive resonance, we first looked at the salience of climate change in legacy media (Fig 2A)—a prerequisite to study the resonance of different venues in this issue. Fig 2A shows that legacy media reported frequently on climate change. However, two observations are worth mentioning: first, conservative media are responsible for only a third of the articles in our sample (although they constitute more than half of our sample). The other two thirds of the articles on climate change are published by left-leaning media and media without a clear political profile (hereinafter referred to as “other media”). Second, the *volume of reporting on climate change* decreased in both media categories over the course of the two years analyzed as indicated by the sloping regression line in Fig 2A.

### 3.1 Continuous resonance

#### Frame resonance

We speak of a continuous frame resonance if the Jensen-Shannon divergence (JSD) becomes significantly smaller in the two-year period. This would indicate a convergence of online and offline agendas as we calculated the thematic divergence between websites of climate change skeptics and legacy media. However, the trend lines in Fig 2B and the regression models reported in Table 5 show that there was no significant decrease of the JSD for both conservative and other legacy media. As the coefficients indicate, the distance between online and offline agendas remain almost the same over the whole time period. Therefore, neither of the two legacy media types can be attributed a continuous thematic resonance.

**Table 5:** Regression coefficients (β) for divergence measures.

	Thematic resonance		Positional resonance		Actor resonance	
Media	Conservative	Other	Conservative	Other	Conservative	Other
Constant	0.3467 (0.0176)	0.3137 (0.0162)	0.0750 (0.0044)	0.0795 (0.0042)	0.0502 (0.0073)	0.0446 (0.0074)
Time	0.0003 (0.0013)	-0.0006 (0.0012)	0.0005. (0.0003)	0.0005 (0.0003)	-0.0001 (0.0005)	0.0003 (0.0005)

Standard errors in parentheses. *** p < 0.001, ** p < 0.01, * p < 0.05,. p < 0.1.

Beyond, a comparison between conservative and other media reveals that there is no indication that conservative media are more open for the skeptics’ frames. In contrast Fig 2B even indicates that the Jensen-Shannon divergence (JSD) between the aggregated online and offline frame agendas is slightly bigger for conservative media than for other media. This means that the climate change framing in left-leaning media and media without a clear political profile is, overall, closer to that of the climate change skeptics on the web compared to that of conservative newspapers and magazines which clearly contradicts the expectations about the special role of right-wing media in giving voice to climate skeptics as regards frames.

#### Positional resonance

With regard to positional resonance, the green trend line with a positive incline in Fig 2C indicates that climate change skeptics became more radical over time. However, the degree of verbal radicalization is marginal (β = 0.0005) and only weakly significant (p = 0.0687) as the coefficients in Table 5 show. Nevertheless, every tenth sentence on an average skeptic’s website explicitly expresses a skeptical position on climate change. The traditional media, however, remain unaffected by this development as the blue and red lines in Fig 2C indicate. The proportion of skeptical sentences is almost identical for both the legacy media categories and remains low over the entire time period. Accordingly, it is no surprise that the divergence between online and offline spheres reported in Fig 2D increases over time. In case of the conservative media, the increase (β = 0.0005, se = 0.0003) is even weakly significant (p = 0.0904). Despite their radicalization, the climate change skeptics have therefore not succeeded in provoking a continuous resonance in legacy media regards their positions.

#### Resonance as regards participants of a debate

As Fig 2E shows, there is no increase in the visibility of skeptical actors in legacy media over time (blue and red line). As the average share of 2% shows, legacy media hardly give skeptical actors a platform, regardless of the media’s political profile. At the same time, there is no increase in the number of skeptical actors on the skeptics’ websites (green line in Fig 2E). As a result, the divergence between online and offline did not change significantly over the period examined (Fig 2F, coefficients in Table 5). This is true for both conservative and all other media over the whole two years. This means that there is no convergence of the discourses of the skeptical counter-movement and the dominant public discourse as presented in legacy media. Thus, there are no continuous resonance effects here either.

Overall, no continuous discursive resonance can be identified. There is no evidence that German legacy media are increasingly following climate skeptics over the course of time. Interestingly, hardly any difference is found between conservative and other legacy media as regards frames, positions and actors, with some counter-intuitive exceptions as regards the framing of the issue. For the German conservative media, we can therefore not confirm what has been shown for their US counterparts—that conservative media in general are allies to climate change skeptics. German legacy media do not seem to offer a platform for climate change skeptics.

### 3.2 Selective resonance

**Fig 3:**
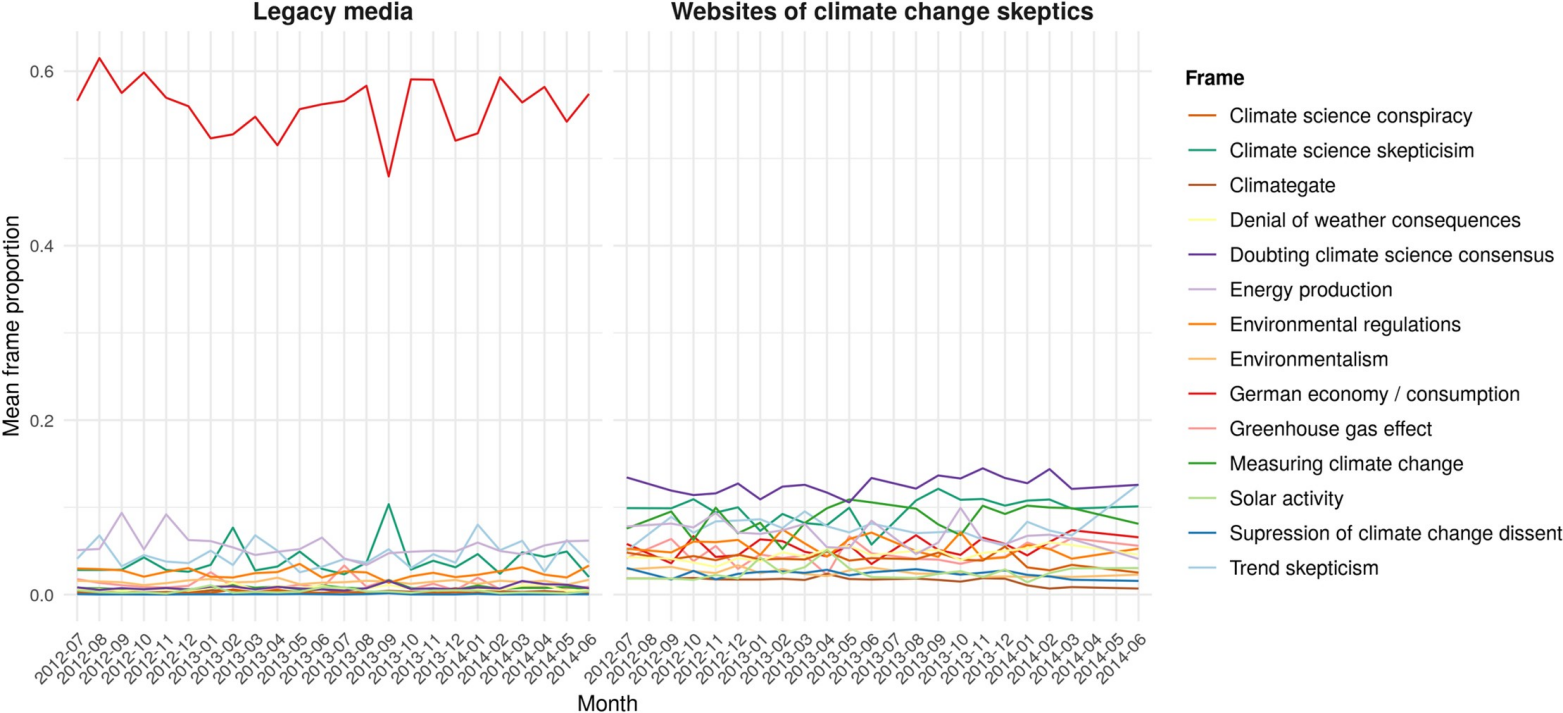
Topic proportions in legacy media and online. Mean topic proportions over time in legacy media and on websites of climate skeptics.

The lack of continuous resonance between legacy media and climate skeptics in Germany as regards frames, positions and actors, results in a debate that is fragmented. On a thematic dimension, this fragmentation becomes visible in Fig 3. It makes clear that German legacy media frame the issue of climate change almost exclusively in terms of the German economy/consumption patterns. This one frame captures most of the mainstream debate, whereas climate skeptics employ a variety of frames with “doubting the climate science consensus,” “climate science skepticism,” and “measuring climate change” being the most prominent. On a positional dimension, this fragmentation is shown in the radicalization of climate skeptics’ communication that is not at all reflected in the mainstream debate (Fig 2C and 2D). In sum, climate skeptics still form a separated counter public in Germany in the time of analysis, that is occupied with its own frames, radicalizes its positions and is not taking-over the economy frame dominating the mainstream debate. The mainstream public, in contrast, is occupied with discussing the economic consequences of climate change while largely ignoring the skeptics’ frames, positions and actors.

Does this, however, mean that German legacy media are immune to climate skeptics’ frames, positions, and allies, or do we find indicators of selective resonance among the different venues? Our data indicate that, in September 2013, selective resonance might have occurred. In this month, the Jensen-Shannon divergence was noticeably low, which indicates thematic resonance (blue dot in Fig 2B). As shown in Fig 3, the frame, “climate science skepticism,” peaked in the legacy media in this particular month, reducing the attention on the dominating frame as regards the German economy, both in the conservative as well as in the other legacy media. This thematic resonance was accompanied by an above average share of skeptical sentences in the legacy media (Fig 2C).

Looking at the articles that have both a high probability of the “climate science skepticism” frame and a high proportion of skeptical sentences, it can be seen that their publication was triggered by two events: the publication of the final draft of the 5^th^ assessment report of the Intergovernmental Panel on Climate Change (IPCC) and the publication of an oceanographic study in the journal *Nature*. While the assessment report contains some more conservative projections than previous versions, the Nature study [85] addresses the lower than predicted temperature increases in recent decades. Both aspects—the IPCC’s supposed failure/fraud and the plateau in the temperature curve—can be regarded as critical events, changing visibility structures within the climate change debates. The skeptics have successfully used these events to put forward their alleged counter-evidence for climate change referring to the news value “surprise”.

Apparently, these scientific publications have opened a window of opportunity for skeptics’ frames and positions to reach out to legacy media’s agenda. A closed-up reading of the relevant articles, however, shows that such punctual visibility of skeptics’ frames and positions in the legacy media have been opposed by the journalists themselves: they criticize skeptics’ interpretations and, thus, accompany this selective resonance with their own critical examination of skeptics’ arguments.

## 4. Discussion

In this article, we examined how different venues—the online venue of climate change skeptics and the mainstream discourse—resonate. Given that counter-movements are by definition oriented towards the mainstream of political debates [8], this paper has investigated the opposite relationship, more challenging from the point of view of democratic theory: do the online counter-movements of climate skeptics resonate in traditional media? We have studied three types of discursive resonance: the media’s adoption of climate change skeptics’ frames (thematic resonance), the inclusion of skeptical positions (positional resonance), and the mentioning of skeptical actors (actor resonance). In addition to this, we have distinguished continuous from selective resonance patterns.

As a first major result of our study, we find no evidence for continuous resonance. We find neither an increase of skeptical frames in the legacy media’s coverage, nor an increase of skeptical positions or actors. Second, however, this does not mean that skeptical voice are invisible in traditional media, as resonance may occur only selectively at single points in time. Our data reveal that specific events—in our case, the publication of scientific reports—can open a small window of opportunity that sees skeptical frames resonate to a greater degree in the media. While even in these cases skeptical positions remain contested by the media, their mere inclusion serves to ratify their views as legitimate contributions to the debate. This can be seen by counter-movements as a first step towards greater possibilities of participation in political debates.

Third, the ideological profile of newspapers and magazines in Germany did not turn out to be relevant: Conservative legacy media in Germany did not give greater resonance to climate skeptical voices. This strongly contrasts with the US, where conservative outlets play an important role in amplifying climate skepticism and are part of the “climate change denial machine” [23 p147]. This finding also underlines the necessity to move research beyond the US context to better understand the role played by different context factors. In Germany, for example, the landscape of traditional media is substantially less polarized than in the US, and–contra the two component theory—journalists across the political spectrum appear to display no difference in their orientation towards news factors. However, while there is no qualitative difference between media outlets in terms of news factor emphasis, there is a telling quantitative distinction between them: while our sample includes more conservative than centrist or left-leaning media, climate change is much less salient in them. Lacking established skeptical actors on the national stage they could use as opportune witnesses in their reporting, conservative media resort to downplaying the issue by allocating less space to it.

How can we explain the lack of continuous resonance in the German case? News value theory hints at three factors (e.g., [41]). First, the network of German climate change skeptics is rather weak. Most of the web pages in the sample are from foreign actors, in particular from the United States, and included in the German issue network by German skeptics. However, but neither the scope nor the language of these actors is necessarily German, and, as such, they are no points of reference for journalists, whose reporting is primarily oriented towards domestic politics. In news value terminology, they lack both the “closeness” and the “prestige”. Second, German skeptics lack prominent speakers, whose status would guarantee continuous resonance. Third, the climate skeptical discourse is largely uncoupled from the policy cycle and its coverage by the media. Whereas news outlets followed the political that revolved around the economic aspects of climate change, skeptics discussed unrelated, more fundamental questions (e.g., whether it occurs or whether one can trust climate scientists), which did not fit into the relevance structure of news reporting. This leaves few options for climate skeptics and their resonance opportunities are mostly selective: without prestigious actors and disengaged from the policy cycle, they rely on event-driven news factors. The “surprise” news factor becomes an important element, and our study shows that skeptics rely on external events like the publication of scientific reports that—from a mainstream perspective—reveal surprising findings.

Our results point out that German legacy media have not played a prominent role in spreading the ideas of climate skeptics. On the contrary, they seemed to fulfill their task of informing the public about climate change in accordance with the scientific consensus (e.g., [27]), and criticized the climate skeptical positions on which they reported, as our qualitative case study shows. While this conforms to the normative standards associated with journalism, the media largely reduced their climate change coverage to the economic aspect, neglecting its political dimension or its international scope. This one-sided focus may hamper the ability to act politically and actually solve the climate crisis.

Moreover, the media’s exclusionary practice towards the skeptical counter-movement has its drawbacks: those supporting the counter-positions might well develop a feeling of alienation and misrepresentation. As a consequence, these people may turn to alternative digital information sources, while their distrust of traditional media increases—a trend that has occurred in recent years in Germany on the political far-right. The media’s decision to refrain for instance from questioning the scientific consensus might well result in losing their role as legitimate gatekeepers in the debate in the eyes of some parts of the population.

Do our results mean that the engagement of counter-movements’ online has no effect? Although skeptics lack a continuous resonance on legacy media agendas, they still have a selective one. Counter-movements’ online communication serves as a “reservoir of ideas” from which they can draw as soon as a window of opportunity opens [12]. However, further research needs to show whether counter-movements’ online communication fulfills more functions. It might be that climate skeptical ideas and positions are disseminated without the help of traditional media. This raises the question of diffusion patterns of climate skepticism and the role played by traditional media, digital outlets, blogs and social media—and how they differ between countries. And depending on the national context, can skeptics bypass the media and directly influence politics and, in this way, gain legitimacy and a wider access to citizens?

Beyond this, future research needs to dig deeper into the conditions under which media resonance occurs. To further understand continuous resonance and the relevance of the news factor “prominence/elite,” Germany would certainly be an interesting case. Here, the recent electoral gains of the right-wing “Alternative für Deutschland” (AfD) have also brought some climate change skeptics into parliament, which in turn might help skeptical voices to become more prominent in traditional media. In addition, more research is needed to understand which specific types of events and conditions make selective resonance more likely. In our research, climate skeptics have relied on external events to promote their ideas. However, can counter-movements create events themselves to advance their agenda (see [86])? Knowledge about these events and conditions might help make journalists more sensitive in their reporting. Finally, more comparative research is needed to gain a better understanding of digital discourse strategies employed by climate skeptics and their resonance in media coverage. Our study already shows that the patterns we observe in Germany are not comparable to the ones in the US. We thus need to systematically study context factors.

Finally, there are a number of limitations that we need to address: First, we speak of the resonance of climate skeptical discourse in the media. Statistically, this corresponds to correlations models and we are aware that we cannot draw conclusions about causal relationships in a strict sense. Particularly with regard to frames, it might be climate skeptics whose discourse is influenced by the mainstream debate covered by the media. Second, empirically, the distinction between frames and positions is not as clear as presented by the theory. Frames define the relevance structure of an issue, regardless of the position of the actors. However, the results of our topic model suggest that some frames are aligned with a specific position (e.g., the denial of weather consequences reflects the skeptical position). At the same time, none of the frames is solely used by one side of the debate [12], and we are confident that our analysis captures the three crucial components of political debates: frames, positions, and actors. Third, our named-entity approach did not allow us to distinguish between speakers and addressees. However, this distinction is important to assess the role of the actors in the media reports. Beyond this, the automated approaches failed to reveal how journalists reacted to an increase in skeptical frames and positions. Here, only a qualitative case study can show journalists’ critical reactions. To this end, additional methodological work is needed.

## Supporting information

S1 Appendix Starting points for the snowball-sampling of websites. (PDF)

S2 Appendix List of German legacy media. (PDF)

S3 Appendix Topic labels and top words. (PDF)

S4 Appendix Performance indicators for the semi-automated classifier. (PDF)
