# Supplementary material for: How climate change skeptics (try to) spread their ideas: Using computational methods to assess the resonance among skeptics’ and legacy media
Source: PLoS One. 2020 Oct 5;15(10):e0240089. doi: 10.1371/journal.pone.0240089 (PMC7535043; doi:10.1371/journal.pone.0240089)
Supplement: S1 Appendix — (PDF) [file pone.0240089.s001.pdf]

S1 Appendix

Starting points for the snowball-sampling of websites. Actors are from the climate skeptical counter-movement in Germany

Actor	Start URL
Analyse+Aktion	http://astrologieklassisch.wordpress.com/tag/klimawandel
EIKE - Europäisches Institut für Klima und Energie	http://www.eike-klima-energie.eu
Klimaüberraschung	http://www.klima-ueberraschung.de
Klimaskeptiker	http://www.klimaskeptiker.info
