# Supplementary material for: How climate change skeptics (try to) spread their ideas: Using computational methods to assess the resonance among skeptics’ and legacy media
Source: PLoS One. 2020 Oct 5;15(10):e0240089. doi: 10.1371/journal.pone.0240089 (PMC7535043; doi:10.1371/journal.pone.0240089)
Supplement: S2 Appendix — (PDF) [file pone.0240089.s002.pdf]

S2 Appendix

List of German legacy media

Newspaper/magazine	Conservative	Number of articles in sample
Bild	x	33
Bild am Sonntag	x	15
Der Spiegel		88
Die Welt	x	347
Die Zeit		175
Frankfurter Allgemeine Zeitung	x	622
FAZ am Sonntag	x	101
Financial Times Deutschland (until 12/2012)	x	47
Focus	x	43
Frankfurter Rundschau		466
Handelsblatt	x	172
Stern		40
Süddeutsche Zeitung		1,225
Taz, die Tageszeitung		622
Welt am Sonntag	x	115
