# Supplementary material for: How climate change skeptics (try to) spread their ideas: Using computational methods to assess the resonance among skeptics’ and legacy media
Source: PLoS One. 2020 Oct 5;15(10):e0240089. doi: 10.1371/journal.pone.0240089 (PMC7535043; doi:10.1371/journal.pone.0240089)
Supplement: S3 Appendix — (PDF) [file pone.0240089.s003.pdf]

S3 Appendix

Topic labels and top words

Label	Top words
Climate science conspiracy	wissenschaft, klima, unterlag, herzland, wissenschaftl, institut, peer, arbeit, mensch, falsch
Climate science conspiracy	e-mail, freitas, papier-, wissenschaft, mann, klima, mannschaft, versuch, sorg, geschicht
Climate science skepticisim	prof, jahr, bitt, ipcc, co2, wissenschaft, global, klimawandel, klima, erwarm
Climategate	wissenschaft, tagebuch, freitas, aktion, papi, hinweis, rezension, klima, phil, person
Denial of weather consequences	klima, co2, ansteig, erhohen,, verander, erwarm, global, mensch, wissenschaft, wett
Doubting climate science consensus	klima, wissenschaft, global, erwarm, verander, ipcc, wissenschaftl, bericht, polit, forschung
Energy production	energi, wind, leistung, vereinigt, regier, polit, land, wirtschaft, europa, kost
Environmental regulations	pflanz, kohlenstoff, mensch, kohl, umwelt, epa, jahr, emission, welt, zustand
Environmentalism	mensch, natur, bericht, vereinigt, klima, konigreich, umgeb, leb, beispiel, auswirk
German economy/consumption	jahr, deutsch, klimawandel, deutschland, gross, prozent, land, energiew, europa, hoh
Greenhouse gas effect	atmosphar, temperatur, co2, oberflach, erhoht, strahlung, erd, energi, luft, ansteig
Measuring climate change	zeigt, temperatur, dat, jahr, erwarm, modell, bedeut, global, trend, zahl
Solar activity	solar-, klima, sonn, erd, zyklus, aktivitat, wolk, kosmisch, strahl, planet
Supression of climate change dissent	wissenschaft, klima, seeland, aufzeichn, temperatur, niwa, verander, position, bemerk, debatt
Trend skepticism	jahr, eis, erhebt, meer, ansteig, erhohen, global, erwarm, wett, temperatur
