# Supplementary material for: How climate change skeptics (try to) spread their ideas: Using computational methods to assess the resonance among skeptics’ and legacy media
Source: PLoS One. 2020 Oct 5;15(10):e0240089. doi: 10.1371/journal.pone.0240089 (PMC7535043; doi:10.1371/journal.pone.0240089)
Supplement: S4 Appendix — (PDF) [file pone.0240089.s004.pdf]

S4 Appendix

Performance indicators for the semi-automated classifier

	Macro F1			Micro F1
**Language**	**Skeptics**	**Advocate**	**irrelevant/neutral**	**overall**
English	0.65	0.68	0.91	0.83
German	0.92	0.89	0.88	0.85

Reported are the F1 scores (harmonic average of precision and recall; macro F1 for the different categories and micro F1 overall) after the third iteration of active learning. The scores are calculated by comparing the machine coding against the manual coding (gold standard).
